# Implementation of a Suicide Risk Screening Instrument in a Remand Prison Service in Berlin

**DOI:** 10.3389/fpsyt.2018.00665

**Published:** 2018-12-11

**Authors:** Dora Dezsö, Norbert Konrad, Katharina Seewald, Annette Opitz-Welke

**Affiliations:** ^1^Institute of Forensic Psychiatry, Charité University Hospital Berlin, Berlin, Germany; ^2^Research & Development Division, Berlin Prison & Probation Services, Berlin, Germany

**Keywords:** suicide prevention, suicide screening, penal institution, pre-trial detention, prison suicide

## Abstract

In the present study, we examined the effects of implementing the suicide risk screening instrument SIRAS in a pre-trial detention facility for men in Berlin. Within a period of 3 months, all newly arriving prisoners were screened (*n* = 611) by social workers or prison officers. Cases of elevated suicide risk were immediately referred to a psychologist or medical staff the same day. Follow-up over a 6-month period showed that 14% of all incoming prisoners were classified as high-risk individuals. These individuals received significantly more psychological and psychiatric treatment and were significantly more likely to be accommodated in crisis intervention rooms and emergency community accommodation (shared prison cells). In addition, it was found that despite the increased amount of treatment in the high-risk group, the number of specific measures did not increase significantly compared to the pre-implementation phase (*N* = 1,510).

## Background

Prevalence of psychiatric disorders among prisoners is many times higher and suicide is one of the leading causes of death in prison (1–4). The exact rate of suicide varies widely according to the study design, with values most frequently reported at 2- to 10-times higher rate compared to the general population (5, 6). Different studies use different suicide-rate calculations, sometimes with major methodological problems (7, 8). Thus, for example, the presumably large number of unreported and hence unofficial numbers of suicides in the general population is not sufficiently taken into account as a comparison group whereas the number of unreported cases in prison settings is relatively low due to close monitoring. The arising problem is the difficulty to compare the suicide rates of those two groups. In addition, statistics can be distorted as well by varying definitions of suicide; some studies include the overdose of drugs or are unable to include suicidal intentions covered as traffic or household accidents (9).

Despite methodological issues, it can generally be claimed that suicide is a real problem in prison and prisoners face an elevated number of risk factors. Konrad (10) found a 6.5 times higher suicide rate among male prisoners compared to the age-and sex- matched general population (11). Lohner and Konrad (12) found that the characteristics of suicides in pre-trial detention appear relatively homogenous as opposed to detention where those risk factors are more heterogeneous.

For example, a census conducted by the criminologists in the Lower Saxony prison on 1067 cases between 2000 and 2013 (*N* = 1,067) shows that among detained prisoners suicide risk among prisoners increased in several age ranges, younger age groups having a higher risk as opposed to remand prisoners where high risk group prisoners were mainly in the over-40s age group. Similarly, the timing of the suicidal act showed differences between sentenced and remand prisoners (13).

It is widely known that within the prison system, suicides are more common among remand prisoners. Especially in the first days of detention, an increased suicide is generally found (5, 14). A possible explanation is the so-called “confinement shock” (2, 15–17). First incarceration experiences, social deprivation, loss of control and uncertainty characterize the period of pre-trial detention and therefore require stable and pronounced coping skills (18).

With regards to suicidal development according to Plödinger (19), 3 phases can be observed. At deliberation stage (Phase 1), suicidality is considered as a possible solution to the problem. The stage of ambivalence (Phase 2) is characterized by a struggle between life-sustaining and self-destructive impulses, direct and indirect suicide announcements can appear. At the decision-making stage (Phase 3), the decision to take one's life is already made and expressed to the environment in form of apparent relaxation and calmness and should not be misunderstood as improvement (20). Hence, the identification of individuals at risk of suicide requires a lot of attention and sensitivity and is even a greater challenge in daily routine.

One option to deal with this problem is the use of screening tools to be able to detect prisoners at high risk of suicide faster and transfer them to specialized staff accordingly. By identifying risk factors highly associated with suicide screening procedures can be used for all prisoners and detect vulnerable individuals. It is important to emphasize that screening procedures are not designed to replace a professional judgment. In fact it can facilitate to transfer high-risk prisoners for further assessments (21) since when assessing the risk of suicide not only the presence of certain risk factors must be considered but suicidality should also be clarified in a direct, empathetic and open face-to-face conversation (22).

If we want to measure the effects of suicide prevention, we face the problem of small absolute numbers in prison settings. To capture the impact of introducing a screening instrument it is therefore only possible to measure parameters that, according to established literature (1, 2, 8, 23–27) are associated with acute crises and suicidal behavior such as frequency of psychological interventions, psychiatric consultation, referral to inpatient psychiatric wards, use of antidepressant medication and other psychotropic drugs, transfer to high-secure-cells due to acute suicidal tendencies, arrangement of special observations and placement in emergency community accommodation i.e., shared prison cells.

Some of these associated parameters are well-documented in the context of the German prison system in the established literature.

In Germany, prison sentences are usually served in single accommodation. Research shows that most suicides are committed between 7:00 p.m. and 7:00 a.m. (28) and in single accommodation (8). Therefore, in Germany, in the case of suspected suicidality, a so-called “emergency community accommodation” is ordered as a preventive measure to reduce suicide risk. When an “emergency community accommodation” is ordered, the detainee is moved to a community cell with two detainees each. This reduces social isolation and facilitates interpersonal exchange with a roommate. Another measure in the case of suicide suspicion is the order of special observation, in which the staff visits the detainees at regular intervals. Liebling (29) found that people preferred to share a detention room before their suicide attempt, that they experienced isolation and were more frequently in crisis intervention space. An extensive study of 423 suicides (9) found that about two-thirds of the prisoners were in solitary confinement at the time of the suicide.

Although the use of antidepressant medication in the treatment of depression and suicide prophylaxis is controversial in the literature and the media (30), it is assumed, according to status quo in medicine, that antidepressant medication counteracts feelings of tension, insomnia, and depression. Studies indicate a prevalence of 14–95% of mental illnesses in suicide cases in prisons (16, 17, 31, 32). In particular, depressive and psychotic disorders show a strong association with suicidality (33), so it can be assumed that these changes act in the sense of suicide prevention (2).

In the study we used the associated parameters mentioned in this section to follow up the usefulness of the modified suicide screening method SIRAS (11).

## Aims and Hypotheses

The present study aims at examining the impact of the implementation of a suicide risk screening tool in Berlin remand detention. The study continues the work by Dahle et al. (11) using the screening instrument SIRAS and—by measuring the impact on the number of specific interventions during the study period and its targeting to the identified high-risk-group—testing the usefulness of implementing a short suicide risk screening instrument in practice. The screening was implemented with an Experimental Group (EG) of prisoners arriving to the prison facility over a certain timeframe and compared with a Comparison Group (CG) of prisoners arrived prior to the implementation. Both groups were followed up for the subsequent 6 months to test the hypothesis.

The following research hypotheses were examined:

Hypothesis 1: The screening instrument reliably predicts suicide risk.
Hypothesis 2: High-risk inmates receive a significantly higher amount of interventions compared to the non-high-risk group.

2.1 High-risk inmates will receive significantly more psychological interventions.
2.2 High-risk inmates will receive significantly more psychiatric examinations.
2.3 High-risk inmates will receive significantly more often psychopharmacological treatment.
2.4 High-risk inmates will be transferred more frequently in the Crisis Intervention Room (CIR).
2.5 High-risk inmates will be referred significantly more often to inpatient psychiatric treatment.
2.6 Specialized observations are ordered significantly more often in the case of high-risk inmates.
2.7 For high-risk inmates, emergency community accommodation (i.e., shared cells) will be ordered significantly more often in the future.

Hypothesis 3*:* In the Experimental Group (EG) the interventions are more targeted as in the Comparison group (CG). There is no significant difference in the number of interventions between the Experimental Group (EG) and Comparison group (CG).

Through the structured introduction of the screening, our aim was to expand the skills of employees in dealing with suicidality and thus relieve the staff.

## Methods

### Materials

The suicide screening procedure (SIRAS) was used for a period of 3 months between March and May in Berlin pre-trial detention for men.

The instrument is based on the Dutch instrument Screening of Suicide Risk of Prisoners by Blaauw et al. (25). By analyzing the files of 95 detainees who died of suicide, the research group identified 8 risk factors that could be replicated both in UK and US prison settings (34).

Aiming at simplifying the application of the instrument for clinically untrained personnel in German prison settings Dahle et al. (11) conducted a retrospective file analysis of 30 prisoners who died of suicide in the Berlin pre-trial detention. The instrument was validated, optimized and translated in German. For non-clinical use, the two items “Psychosis or Axis II Disorders (DSM-IV)” and “Past Psychiatric Treatment” were removed from the original version. Although these clinical factors are of great relevance, the assessment of these items in the screening process by non-clinical staff is difficult.

In addition, the evaluation process was simplified by recoding. The new threshold of 3 points was determined using an ROC analysis (AUC 0.881, *p* < 0.001, 95% CI from 0.793 to 0.969). The modified version of the screening sheet had a sensitivity of 70% and a specificity of 93%. (11).

The final version of the German Scale for Initial Risk Assessment (SIRAS) contains the weighted items presented in Table 1: age, pre-detention, drug use, previous attempted suicide or self-harming behavior, current suicidal statements, or suicide attempts (35).

**Table 1:** Description of screening items and rating.

**Items**	**Description**	**Yes**	**No**
Age 40+	Aged 40 years or more	1	0
No permanent residency	No permanent residency prior to incarceration	1	0
None or one previous incarceration	None or one previous incarceration	1	0
Multiple misuse of drugs	Biographical consumption of serious drugs (at least one a week) combined with regular consumption of weaker drugs and/or consumption of a greater amount of alcohol and/or medication.	1	0
Known previous suicide attempts or self-harming behavior	Biographical suicidal attempts or intentional self-harming behavior (cuts, intoxication, etc.) are known.	1	0
Suicidal expressions or suicide attempt	Suicidal ideation is expressed during current incarceration or suicide attempts have taken place already.	3	0
Sum			

*With a sum score of three or more the individual should immediately be transferred to a psychologist or medical staff*.

### Procedure

Before the key date of the implementation, the users, namely social workers and prison officers were informed about the theoretical background of the screening and received training for the instrument.

As can be seen in Table 1, the data included in the screening instrument are basic data that are usually collected during the admission process. Thus, the novelty is not the collection itself but the structured form the tool been used and the obligatory presentation to a psychologist or medical staff when a certain cutoff (3 or more points) is reached. From 01.03.2016 to 31.05.2016 the screening was carried out with each new arrival to the prison facility. A group of prisoners who entered the prison during the 3 months prior the study period (01.12.2015–29.02.2016) served as comparison group. Both groups were followed up for the subsequent 6 months. The experimental group was additionally divided according to the screening results into a high-risk group (sum of 3 or more) and the non-high-risk group.

In the Berlin pre-trial detention each newly admitted person goes through a reception routine. As part of a regular admission interview which is carried out by a social worker or prison officer when prisoners arrive outside of office hours, the SIRAS sheet was completed and the result recorded in the digital documentation system. In the case of a positive screening result of three points or more, the person had to be presented to a psychologist or medical staff the same day, who would initiate adequate interventions in case of indication.

### Participants

The sample consisted of all arrivals to Berlin remand prison between March and May 2016. Two exclusion criteria were defined (1) transport prisoners were excluded who, because of their status, did not undergo the routine procedure of pre-trial detention and probably spend only a short time in the prison; (2) those detainees who had been admitted prior to the study period but were temporarily transferred to the prison hospital for health reasons.

The final sample included data from 1,510 male volunteers, the mean age in the comparison and experimental group was 35 years. All the subjects participating were admitted and located in remand prison. Majority were in remand although some of the inmates were already convicted. Table 2 shows descriptive results. The majority of subjects were accused of theft (40.07%), drug offenses (15.43%), and fraud (13.77%).

**Table 2:** Descriptive of study sample.

**Variable**	**Study period**	***N***	**Remand custody**	**Age min–max**	**Mean age**
Comparison group	01.12.15–29.02.16	899	70%	20–97	35.2
Experimental group	01.03.16–31.05.16	611	69%	21–73	35.3

### Data Analysis

Data entry and analysis was performed by the first author who was not associated to the prison but present on-site as a point of contact every week during the study period. The analysis was carried out using SPSS (36). The variables were tested for normal distribution using the Kolmogorov–Smirnov test. In the absence of a normal distribution, non-parametric methods (Kruskal–Wallis test and Chi-squared test) were used, which do not have the assumption of a normal distribution or a similarly large group size. The significance level was set at 5%.

## Results

During the implementation phase, *n* = 834 detainees were admitted to the remand prison facility. In *n* = 223 cases data on suicide screening was missing, resulting in a total sample of *n* = 611 collected screening data, of which *n* = 605 were reliably completed and could be considered in the evaluation. In order to avoid any damage, the questionnaires which were not completely filled out but reached a sum score above three (crucial cut off) were considered for inclusion into the high-risk group, leading to a sample of *n* = 611.

**Figure 1:**
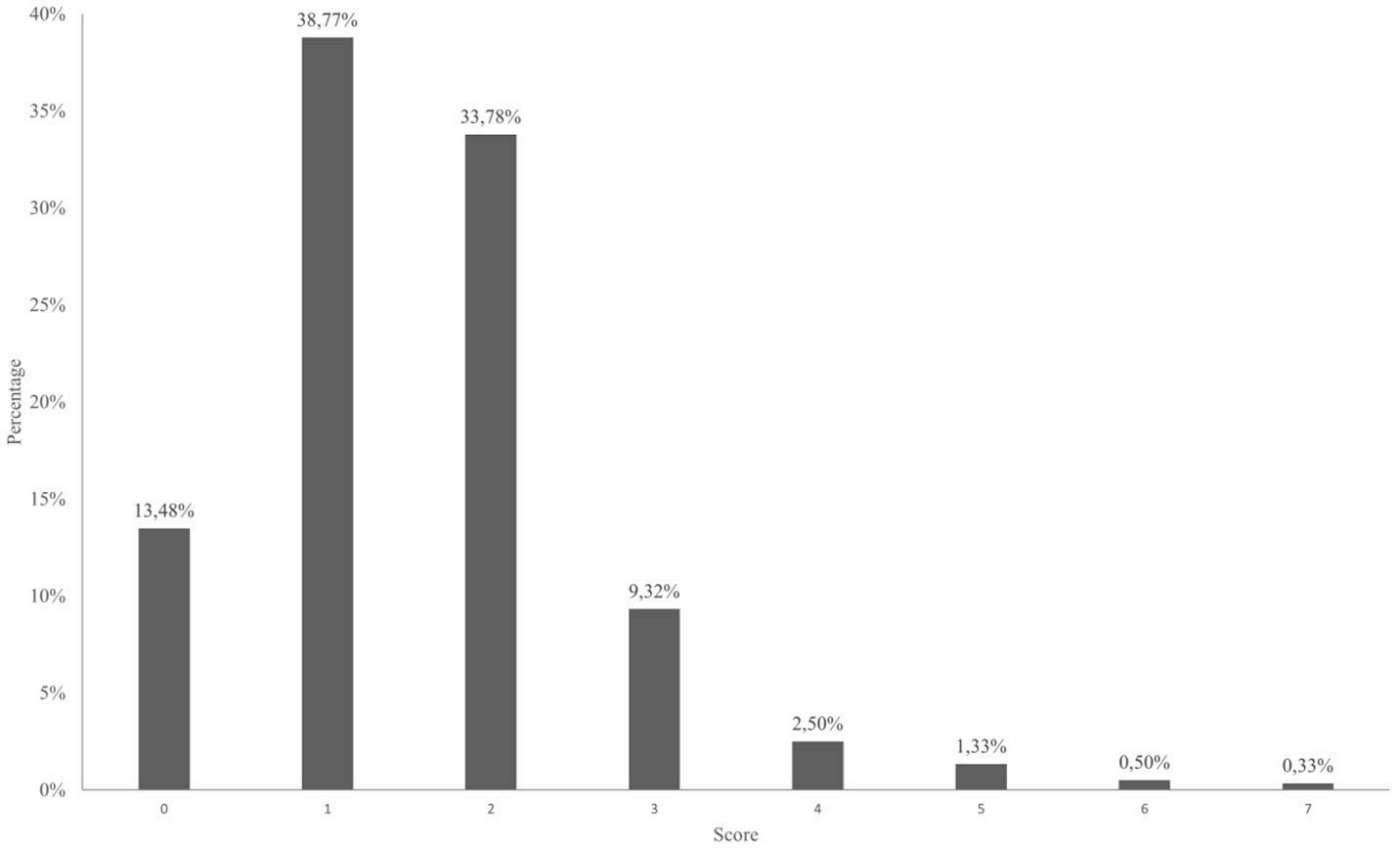
Distribution of sum scores in screening tool for new arrivals during study period.

Sum score ranged from 0 to 7 point, mean score was 1.56, standard deviation was 1.10. The overall result is that 14.21% (*n* = 86) of the subjects met the screening criteria as a person at high-risk of suicide. Thus, 14.21% (*n* = 86) of newly arrested detainees were presented to a psychologist or medical staff on the day of arrival. Looking at the scores in detail (Figure 1), it can be noted that 9.32% (*n* = 56) of the experimental group reached a score of 3 points, 2.50% (*n* = 15) a score of 4 points, 1.33% (*n* = 8) a score of 5 points and < 1% reached 6–7 points (*n* = 3, *n* = 2) (37).

No clear pattern can be identified when comparing the SIRAS score to allegedly committed offense. It can however be established that 39% (*n* = 22) of the detainees with a SIRAS score of 3 and 33% (*n* = 5) of the detainees with a SIRAS score of 4 were detained for alleged theft, while 38% (*n* = 3) of the detainees with a SIRAS score of 5 were detained for alleged drug offenses. Detainees with a SIRAS score of 6 were detained in equal ratios either for alleged causing of bodily injuries, drug offenses, or theft (33% each, *n* = 3). Detainees with a SIRAS score of 7 were detained in equal parts for alleged sexual assault or theft (50% each, *n* = 2).

Hypothesis 1. During the study period, there was no suicide reported in the facility. In *n* = 4 individuals enforceable arrangement was documented (e.g., emergency community accommodation, special observation, crisis intervention room).

**Figure 2:**
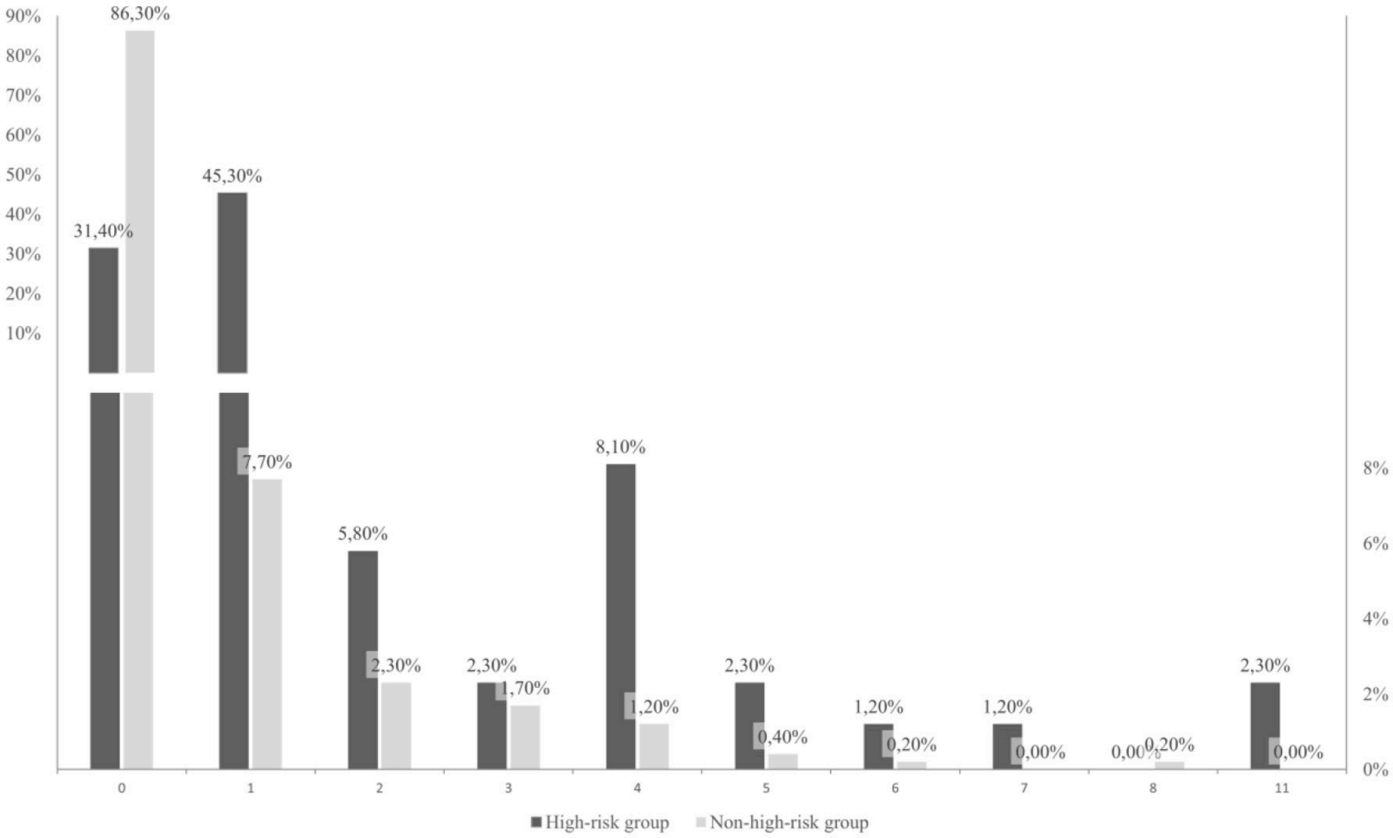
Percentage distribution of psychological interventions between the experimental groups (high-risk and non-high-risk). The chart is using a finer scaling until 10% to display smaller percentages.

Hypothesis 2.1. There were significant differences in the number of psychological interventions (*N* = 605, Kruskal–Wallis test, *p* ≤ 0.01) between the high-risk group and the non-high-risk group. There were no psychological interventions in 31.4% (*n* = 27) high-risk prisoners, instead they received medical attention, potentially because they were admitted after 5 p.m. In 45.30% (*n* = 39), a single psychological intervention was conducted. Two or more psychological interventions were conducted with 23.20% (*n* = 20), with 2 and 4 interviews being most frequent (*n* = 5, *n* = 7) (Figure 2). Looking at the non-high-risk group, it can be observed that 86.30% (*n* = 448) did not receive a single psychological interview, 7.70% (*n* = 40) a single and only 6.00% received 2 or more subsequent interventions (Figure 2).

Furthermore, significant differences in the frequency of the psychiatric consultation (*N* = 605, Kruskal–Wallis test, *p* ≤ 0.01) were observed (Hypothesis 2.2).

Looking closer at the psychopharmacological treatment, 40.70% (*n* = 35) of the high-risk group and 20.62% (*n* = 107) of the non-high-risk group did receive any type of psychopharmacological medication. 22.09% (*n* = 19) of the high-risk group and 8.67% (*n* = 45) of the non-high-risk group received neuroleptic or sedative medication. 23.26% (*n* = 20) of the high-risk group and only 12.91% (*n* = 67) of the non-high-risk group received antidepressant medication.

Data confirms significantly more prisoners in the high-risk group received antidepressant medication (hypothesis 2.3) as opposed to the non-high-risk group (*N* = 605, Pearson chi-squared test = 6.414, *p* < 0.05).

Hypothesis 2.4 could also be confirmed, high-risk individuals were placed significantly more often in the crisis intervention rooms (*N* = 605, Kruskal–Wallis test, *p* = 0.000) (Table 3).

**Table 3:** Rank mean scores of accommodation in crisis intervention cell.

**Variable**	***N***	**Mean rank**
Non-high-risk group	519	299.19
High-risk group	86	325.97

Differences between the high-risk and non-high-risk groups regarding admission to inpatient psychiatric treatment (Hypothesis 2.5) did not exist (*N* = 605, Pearson chi-squared test = 4.229, Exact *p* = 0.099).

Hypotheses 2.6 and 2.7 could be confirmed. The number of special observations (*N* = 605, Kruskal–Wallis test, *p* < 0.01) and emergency community accommodation (*N* = 605, Kruskal–Wallis test, *p* < 0.01) were significantly different between the high-risk group and the non-high risk-group.

Following section presents retrospective analysis of the comparison group (admission between 01.12.2015 and 29.02.2016) to the experimental group. Results confirm Hypothesis 3:

Overall, there were no significant differences given the frequency of psychological interventions (*N* = 1,510, Kruskal–Wallis test, *p* = 0.185) and psychiatric consultations (*N* = 1,510, Kruskal–Wallis test, *p* = 0.881).

Also, there were no significant differences (*N* = 1,510, Pearson chi-squared test = 6.414, *p* = 0.880) in the antidepressant drug prescription. 14.68% (*n* = 132) of the subjects in the comparison group and 14.40% (*n* = 88) in the experimental group received antidepressant medication. 11.34% (*n* = 102) of the subjects in the comparison group and 10.47% (*n* = 64) in the experimental group received neuroleptic or sedative medication.

Significant difference (*N* = 1,510, Pearson chi-squared test = 13.844, *p* < 0.01) was found with regards to admission to inpatient psychiatric treatment. In the comparison group, *n* = 33 out of *N* = 899 people (3.7%) and in the experimental group, *n* = 4 out of *N* = 611 people (0.6%) required inpatient treatment.

There were no significant differences in the arrangements of emergency community accommodation (*N* = 1,510, Kruskal–Wallis test, *p* = 0.747) and specific observations (*N* = 1,510, Kruskal–Wallis test, *p* = 0.280).

## Discussion

By introducing the screening, our aim was to implement a structured course of action for the deliberate handling of suicidality in the remand prison system, without generating a significant, unreasonable additional effort.

In order to test effectiveness of suicide handling we set up the study to analyze the number of suicide related treatments between the risk groups. The dimension of additional effort was analyzed comparing the overall number of treatments before and after the implementation phase of the screening tool.

14.21% (*n* = 86) of the experimental group scored with 3 points or more and were thus classified as high-risk individuals. The comparison of the experimental (EG) and comparison group (CG) showed that there was no significant additional effort during the study period compared to the period before introducing the new screening tool. However, a significant effect was the shift in focus of interventions in favor of the high-risk group. This indicates a more effective use of resources after implementation of the screening tool.

Most interesting is the distribution of the need of psychological interventions and antidepressant medication. It is assumed that a psychologist is able to assess the function and the limits of a screening tool and examine the indication for further intervention. Significantly more subjects in the high-risk group received 2 or more psychological interventions on the basis of clinical criteria and not only because of an elevated screening score. The results from analyzing the experimental group and the comparison group support this hypothesis, since the general number of interventions in the implementation phase (study period) did not increase significantly. One possible explanation for this result is that individuals who were actively requesting psychological interventions or who were very vocal and exposed are often screened before the screening. However, this approach often overlooks individuals who are at risk of suicide. It is probable that the introduction of the screening tool enabled the psychologists to address another clientele, the silent endangered (38).

It was found that high-risk individuals were more likely to be accommodated in an emergency community, crisis intervention cell or to receive specific observations. The accommodation in the crisis intervention room is sometimes necessary to isolate individuals from external stimuli. However, it is also critical to note here that isolation in a phase of acute suicidal tendencies does not always work in the sense of suicide prevention and is sometimes used as a disciplinary measure even for prisoners with pronounced behavioral problems (38). It can only be claimed that it is an indicator that the detainee has become exposed in any way. Nevertheless, the comparison of the comparison- and the experimental group showed that before implementing the screening, more frequent accommodation in the crisis intervention room and in-patient psychiatric accommodation were necessary. One possible explanation could be the early receipt of psychological and psychopharmacological support during the study period of implementing the new tool.

This supported by the results showing that antidepressant drug prescription and other psychopharmacological medications were increased in the high-risk group, but generally no more prescriptions were recorded during the implementation period compared to the period before implementation.

## Clinical Implications

Suicide in jail is influenced by the combination of individual and institutional risk factors (1). Since specific prisons differ widely in terms of various aspects affecting suicidality (type of detention, level of overcrowding, number of professionals, structural conditions, management style, etc.) it is important to emphasize that any transformation or modification of the screening sheet should be accompanied in advance by statistical knowledge. Lohner (39) propose that each institution should set up a risk profile with regards to its individual circumstances or, in view of the scarcity of resources, use at least one screening instrument which, in the developmental sample, resembles that of its own institution.

Future studies should also consider the salutogenic model by Antonovsky (40), and include the nature and the relation of stressors and risk factors and generalized resistance resources (protective factors) in the risk assessment of suicidality.

Also, since the inclusion of interpreters on the day of admission seems to be relatively difficult future studies should target how conducting a suicide screening with non-German-speaking prisoners can be managed.

## Strengths and Limitations of the Study

The main advantage of the study is the prospective design. Collecting pre-defined variables tailored to the requirements of the study is less prone to bias errors and uncovers additional knowledge as opposed to a retrospective design studies (18).

An additional strength of the study is the large sample size and the nature of the sample as a cohort of admissions.

Most research in suicide prevention follows a retrospective design and can thus measure suicidality with fixed outcome events (8). The key limitation of this study comes from the use of associated parameters to measure the risk of suicide. As we opted to gain additional knowledge in comparison to the various retrospective file analyzes (18), the prospective design of this study lacked focus on the outcome event of suicide. In the future, further validation of the suicide screening tool using outcome measures of suicide should be undertaken.

Another limitation of the study comes from its naturalistic design. Some factors (e.g., staffing and prison regimen) are hard to keep stable during the length of the study.

## Conclusion

Finally, with implementing a simple and short screening, various changes in the handling of suicidality in remand prison system were noticeable. There has been a shift in specific interventions toward the high-risk group, while the number of interventions in the period before and during the implementation phase did not significantly increase. In addition, it can be stated with reservations that fewer psychiatric decompensation levels were recorded during the implementation of the tool. Through a structured process, the psychologists were more involved in the admission procedure and could use its expertise more effectively. The screening fulfilled the goal of establishing a structured and cost-efficient course of suicide prevention and thus can support staff in the long run.

## Ethics Statement

This study was carried out in accordance with the recommendations of the Charité's Ethics Committee (Berlin) and in accordance with the Declaration of Helsinki. Since the study relied on basic data already collected by the institution, no separate written consent was necessary by the subjects. The study was conducted in full compliance with the predefined terms of and under the prior approval of the Ethics Commitee of the Charité University Hospital Berlin.

## Author Contributions

DD, AO-W, and NK designed the study. DD collected the data. DD, AO-W, and NK analyzed and interpreted the data. DD and AO-W wrote the initial draft of the manuscript. DD and AO-W had full access to all the data in the study and take responsibility for the integrity of the data and the accuracy of data analysis. KS translated and proof read the manuscript. All authors have contributed to read, and approved the final version of the manuscript.

### Conflict of Interest Statement

The authors declare that the research was conducted in the absence of any commercial or financial relationships that could be construed as a potential conflict of interest.
